# Assessing COVID-19 knowledge, attitudes, and practices among hospital employees: identifying sociodemographic determinants for improved public health strategies

**DOI:** 10.3389/fpubh.2024.1295566

**Published:** 2024-01-17

**Authors:** Layla Aljasim, Nargis Begum Javed, Carlos Cordoba, Haider Alyaseen, Batool Aljasim, Mariam Aljasim, Magdalena Cordoba, Bussma A. Bugis, Mohammed Al-Mohaithef

**Affiliations:** ^1^Almoosa Specialist Hospital, Al Mubarraz, Eastern Province, Saudi Arabia; ^2^Department of Public Health, College of Health Sciences, Saudi Electronic University, Dammam, Saudi Arabia; ^3^Division of Plastic and Reconstructive Surgery, Center Hospitalier de I’Universite de Montreal (CHUM), Montreal, QC, Canada; ^4^Almoosa Specialist Hospital, Albatalia, Hufuf, Eastern Province, Saudi Arabia; ^5^Alahsa Health Cluster, Saudi Arabia Ministry of Health, Al Mubarraz, Eastern Province, Saudi Arabia; ^6^Saudi Arabia Ministry of Health, Al Mubarraz, Eastern Province, Saudi Arabia; ^7^School of Physical and Occupational Therapy, McGill University Health Center, Montreal, QC, Canada; ^8^Department of Public Health, College of Health Sciences, Saudi Electronic University, Riyadh, Saudi Arabia

**Keywords:** attitude, COVID-19, hospital employees, knowledge, practice

## Abstract

**Background:**

The working environment of healthcare institution during pandemic puts all hospital employees at high-risk of being exposed to contagious infections. An individual’s behavior and response are largely determined by their level of knowledge, attitudes and practices (KAP) toward a disease. Therefore, the present study aimed to evaluate and assess the KAP toward COVID-19 among hospital employees working in various positions and to identify the sociodemographic determinants associated with the level of KAP.

**Methods:**

A cross-sectional survey was conducted from July 1 to July 15, 2020 in Almoosa Specialist Hospital, Alhasa, Eastern Province, Saudi Arabia in which 221 hospital employees with varied job titles participated. The data for demographics and history of COVID-19 exposure, KAP related to COVID-19 spread and prevention were collected online using a web-based platform (Survey Monkey). Student’s *t*-test/One-way ANOVA were used to compare total mean and standard deviation of KAP scores with demographic profiles and history of exposure.

**Results:**

89.1% employees knew that COVID-19 virus is mostly transmitted from human-to-human, and 76.0% employees acknowledged droplet transmission. 64.7% employees preferred to take a sick person with unconfirmed COVID-19 to a health facility. Physicians had higher knowledge scores for COVID-19 infection and non-medical employees had the lowest scores (7.47 ± 1.23 and 6.47 ± 1.44, respectively). Nurses had the highest practice scores and non-medical employees lowest practice score (6.16 ± 0.74 and 5.37 ± 1.14, respectively). Attitude scores were similar among all the employees. All employees reported an increase in hand-washing frequency and physical contact avoidance.

**Conclusion:**

The study results revealed socio-demographic factors; level of education, nationality, and field of service are associated with COVID-19 KAP. The study highlights that there is a gap in the level of knowledge about COVID-19, especially among nonmedical employees. Targeted interventional programs need to be planned and implemented to improve COVID-19 awareness among non-medical employees.

## Introduction

At the end of December 2019, Wuhan City in China witnessed an unexpected increase in cases of a disease with pneumonia-like symptoms. The World Health Organization (WHO) termed the disease “Coronavirus disease 2019 (COVID-19)” in February 2020, and by March, it was declared a pandemic (1). Patients with COVID-19 were observed to have common symptoms, such as fever, cough, shortness of breath, sore throat, and loss of taste and smell (2).

Saudi Arabia identified its first case of COVID-19 on March 2, 2020 (3). In Saudi Arabia, the incidence rate of COVID-19 on July 5, 2020 was 11%, and the overall mortality rate was 5.7/100,000. In the Eastern Province of Saudi Arabia, the point prevalence of COVID-19 on July 5, 2020 was 450.5 per 100,000, and the overall mortality rate was 3.7 per 100,000 (4). The government of Saudi Arabia provided free treatment to all patients, regardless of the severity of their disease. It is fair to say that prior experience dealing with Middle East Respiratory Syndrome (MERS) in 2012 helped the government draft policies and measures to combat this pandemic (5).

During the pandemic, healthcare providers faced the significant challenge of balancing their enormous patient loads, keeping up to date with constantly changing information and knowledge about the disease, and providing a high level of patient care while ensuring safety of their patients and workforce (6). Healthcare workers are the strength of any healthcare system, and a healthy and well-trained workforce is a basic requirement for the community’s health safety.

The support of healthcare workers, such as nurses, is a major requirement for direct patient care. During the pandemic, allied healthcare professionals were also required to assist in the seamless functioning of the hospital by providing patient care while remaining in the background. These employees were exposed to various threats, such as pathogens, stress, psychological distress, and exhaustion due to long working hours during the pandemic (7). The lack of knowledge about infectious disease among healthcare workers and paramedics may contribute to delays in diagnosis and management, eventually leading to the rapid spread of disease. It is not just the paramedics and medical professionals; all the employees of the hospital were at increased risk of exposure to the contagion, as they worked in an environment that had an increased number of confirmed and yet to be diagnosed COVID-19 cases.

The WHO recommends the use of contact and droplet precautions, as well as personal protective equipment (PPE), while treating COVID-19 patients to prevent the transmission of disease in the healthcare setting. Proper use of PPE includes making the correct selection of PPE, depending on the environment, followed by properly wearing, removing, and disposing of the PPE. It is also essential to educate employees on using PPE properly, patient screening, and the use of masks as per the WHO guidelines and those of the Centers for Disease Control and Prevention (8), World Health Organization (9, 10).

An individual’s behavior and response are largely determined by their level of knowledge, attitudes and practices (KAP) toward a disease (11). According to KAP theory, “Knowledge is the foundation of behavior change, and belief and attitudes are the driving force of behavior change” (12). Many studies from Saudi Arabia have assessed the knowledge, attitudes and practices of healthcare workers (13–18), medical students (19), and paramedics (20) in different regions. However, only one study from Riyadh has assessed the level of KAP in different employees of a hospital (21).

There is a lack of comprehensive information regarding the level of COVID-19 KAP among different types of hospital employees from Eastern Province, Saudi Arabia. This lack of information among employees concerning COVID-19 knowledge, recommendations, and protocols can have grave consequences.

Therefore, the present study aimed to evaluate and assess the KAP toward COVID-19 among hospital employees working in various positions and to identify the sociodemographic determinants associated with the level of KAP. The present study will assist in identifying healthcare institution population sectors that need more attention to prevent the transmission of disease. This study may contribute to formulating future strategies for the effective management of similar public health calamities.

## Materials and methods

A cross-sectional study was conducted from July 1 to July 15, 2020 in Almoosa Specialist Hospital, Alhasa located in Eastern Province of Saudi Arabia. The study population comprised of all hospital employees. The study protocol was reviewed and approved by the Institutional Research Board of Almoosa Specialist Hospital, reference number: ARC-20.12.06. A convenience sampling method was used to enroll the employees. The inclusion criteria were hospital employees with different job titles and willing to participate in the survey.

### Sample size calculation

The sample size was calculated using the online software “Raosoft sample size calculator.” A sample size of 220 was obtained by taking a population size of 2000 (Hospital employees), precision of 5%, confidence level of 95%, and response distribution of 80% from the previous study (22).

### Questionnaire

An online, close-ended questionnaire was developed from previous studies with modifications to assess the knowledge, attitudes and practices of hospital employees toward COVID-19 (11, 22). The questionnaire was evaluated by three experts in the field of epidemiology for its clarity, representativeness, and validity. As per the experts’ suggestion, two knowledge questions and one attitude question were revised for representativeness and clarity.

A pilot study was conducted on 20 hospital employees; these employees were later excluded from the survey. Cronbach’s alpha test for internal consistency of items for knowledge was 0.869. The employees reported that the questions were clear and easy to understand. The employees took approximately 15 min to complete the questionnaire.

The questionnaire had four sections: demographics and history of exposure to COVID-19 and knowledge, attitudes and practices about the spread and prevention of COVID-19. The demographic questions included age, gender, nationality, marital status, number of children, education level, and profession, while the history of exposure questions were “Have you tested positive for COVID-19 infection,” “Has anyone living with you tested positive for COVID-19” and a history of self-quarantine.

There were 10 knowledge questions, which were scored dichotomously as “Correct response/Good knowledge” = 1 and “Incorrect response/Poor knowledge” = 0; six attitude questions were scored as “Good attitude” = 2, “Intermediate attitude” = 1 and “Poor attitude” = 0 or dichotomously as “Good attitude” = 1 and “Poor attitude” = 0; and seven practice questions which were scored dichotomously as “Yes” = 1 and “No” = 0.

The questionnaire was prepared in English and then translated into Arabic because some of the employees were familiar with Arabic. Both the English and Arabic versions of the questionnaire were made available to the participants.

### Data collection

The data were collected online using a web-based platform (Survey Monkey). The researcher contacted the employees and described the study objectives of the survey. Verbal informed consent was obtained from all the participants and the survey link was sent by mobile device for self-administration of the questionnaire.

### Statistical analysis

The Statistical Package for Social Sciences (IBM SPSS Statistics Version 24) was used for the statistical analysis. The employee’s responses for the demographic profile, history of exposure, knowledge, attitudes, and practices are shown as frequency and percentages in the result section. The demographic profile and history of exposure were compared with total mean and standard deviation of knowledge, attitudes, and practice scores using a Student’s *t*-test/One-way ANOVA as these variables showed a normal distribution. A *p* value of less than 0.05 was considered significant.

## Results

A total of 221 hospital employees completed the survey. The demographic distribution of the hospital employees is shown in Table 1. The majority of the employees were in the age range of 31–40 years (*n* = 109; 49.3%); there were 133 females (60.2%) and 170 non-Saudis (76.9%). The majority of employees who participated were graduates (*n* = 178; 80.5%) and nurses (*n* = 118; 53.4%).

**Table 1:** Demographic characteristics and history of exposure to COVID-19 infection in study population.

Characteristics	Number (*n* = 221)	Percent (%)
Age group		
21–30	68	30.8
31–40	109	49.3
41–50	34	15.4
51–60	10	4.5
Gender		
Male	88	39.8
Female	133	60.2
Nationality		
Saudi	51	23.1
Non-Saudi	170	76.9
Marital status		
Single	78	35.3
Married	143	64.7
Children		
Yes	95	43.0
No	126	57.0
Education level		
High school	19	8.6
Graduate	178	80.5
Postgraduate	24	10.9
Profession		
Non-medical	45	20.4
Para-medical	22	9.9
Nurses	118	53.4
Physicians	36	16.3
Have you tested positive for COVID-19 infection		
Yes	11	5.0
No	210	95.0
Do any one who living with you in your home tested positive for COVID-19 infection		
Yes	44	19.9
No	177	80.1
Have you self-quarantined or told to self-quarantine yourself		
Yes	61	27.6
No	160	72.4

### Knowledge about COVID-19 infection among hospital employees

The distribution of responses to the knowledge questions is shown in Table 2. Most of the employees (*n* = 180; 81.4%) did not know the definition of “quarantine”; however, 197 (89.1%) employees knew that the COVID-19 virus is most commonly transmitted from human to human, and 168 (76.0%) employees knew it is transmitted through droplets. The majority of the hospital employees (*n* = 174; 78.7%) knew that coughing, sneezing and/or talking can spread infection, and 150 (67.9%) employees correctly identified seven or more symptoms associated with COVID-19. Furthermore, 194 (87.8%) employees provided correct responses regarding the duration of quarantine and the isolation period, and 52 (23.5%) employees answered correctly regarding the duration of handwashing with soap and water to prevent COVID-19.

**Table 2:** Distribution of responses for knowledge questions.

Knowledge question	Number (*n* = 221)	Percent (%)
K1. Quarantine is preventing the movement of those who have confirmed medical diagnosis of communicable disease?		
True	180	81.4
False^*^	41	18.6
K2. COVID-19 virus is most commonly transmitted by: (Choose one option)		
Animal-to-human	4	1.8
Human-to-human^*^	197	89.1
Environment-to-human	15	6.8
Do not know/not sure	5	2.3
Correct response	197	89.1
Incorrect response	24	10.9
K3. COVID-19 virus is commonly transmitted through: (Choose one option)		
Aerosol	44	19.9
Droplets^*^	168	76.0
Do not know/not sure	9	4.1
Correct response	168	76.0
Incorrect response	53	24.0
K4. Person infected with COVID-19 can spread infection during close contact by: (Select all apply)		
Cough + Sneezes + or/Talk^*^	174	78.7
Cough	24	10.9
Sneeze/Talk	14	6.3
Cough + Sneezes + Talk + Smiles	7	3.2
Do not know/not sure	2	0.9
Correct response	174	78.7
Incorrect response	47	21.3
K5. What are the common symptoms of COVID-19 infection? (Select all apply)		
Fever	217	98.2
Cough	207	93.7
Difficulty breathing	187	84.6
Fatigue	135	61.1
Muscle pain	125	56.6
Severe Headache	161	72.9
Loss of smell	157	71.0
Sore throat	183	82.8
Vomiting	85	38.5
Diarrhea	146	66.1
Poor knowledge (<7 symptoms)	71	32.1
Good knowledge (≥7 symptoms)	150	67.9
K6. Isolation is preventing the movement of those who have confirmed medical diagnosis of communicable disease?		
True^*^	196	88.7
False	25	11.3
K7. Can the COVID-19 virus spread through close contact with infected persons like those caring for each other and/or living together?		
Yes^*^	209	94.6
No	5	2.3
Do not know/Not sure	7	3.1
Correct response	209	94.6
Incorrect response	12	5.4
K8. Individuals at higher risk with COVID-19: (Select all apply)		
Advanced age	203	91.9
Cancer	163	73.8
Cardiovascular disease	174	78.7
Chronic respiratory disease	211	95.5
Diabetes	160	72.4
High blood pressure	141	63.8
Poor knowledge (≤4 risks)	76	34.4
Good knowledge (>4 risks)	145	65.6
K9. How long should a person suspected/confirmed with COVID-19 be quarantined/isolated		
7 days	9	4.1
14 days^*^	194	87.8
21 days	11	5.0
More than 21 days	1	0.4
I do not know/Not sure	6	2.7
Correct response	194	87.8
Incorrect response	27	12.2
K10. Hand washing with soap and water for COVID-19 virus prevention should be done at least for		
10 s	5	2.3
20 s^*^	52	23.5
30 s	24	10.9
40 s	74	33.5
50 s	2	0.9
60 s	64	28.9
Correct response	52	23.5
Incorrect response	169	76.5

^*^represent correct response; Knowledge score: Correct response—1, Incorrect response—0; Good knowledge—1, and Poor knowledge—0.

### Attitude toward COVID-19 infection among hospital employees

The distribution of responses to the attitude questions is shown in Table 3. Of the total, 122 (55.2%) employees thought that they were likely to develop a COVID-19 infection; most of the employees (*n* = 143; 64.7%) preferred to take a sick person with presumed COVID-19 to a health facility. Furthermore, 98 (44.3%) employees reported that they would visit a person who had recovered from COVID-19 infection; however, most of the employees (*n* = 217; 98.2%) reported that they had not participated in funeral or burial ceremonies in the past 3 months. However, 98 (44.3%) employees reported that they would accept ways of holding a funeral or burial that did not involve touching and washing the corpse. The majority of employees (*n* = 203; 91.9%) reported accepting vaccination to prevent COVID-19 infection.

**Table 3:** Distribution of responses for attitude questions.

Attitude question	Number (*n* = 221)	Percent (%)
A1. How likely do you think you will develop COVID-19 infection?		
Unlikely	33	14.9
Neither likely nor unlikely	66	29.9
Likely	122	55.2
A2. What would you do if you suspect someone in your family has COVID-19?		
Take care at home	23	10.4
Avoid all physical contacts	55	24.9
Take person to health facility/Call hospital/MOH/COVID-19 phone hotline number	143	64.7
A3. Would you visit someone after they recovered from COVID-19?		
Do not know/Not sure	35	15.8
Yes	98	44.3
No	88	39.8
A4. If a family member died, would you accept other ways of funeral/burial that would NOT involve the touching or washing of the dead body?		
Yes	98	44.3
No	60	27.1
Do not know/Not sure	63	28.5
A5. Did YOU participate in a funeral/burial ceremony in the past 3 months (90 days)?		
Yes	4	1.8
No	217	98.2
A6. If there was an approved vaccine that could prevent COVID-19, would you accept it for yourself and your family?		
Yes	203	91.9
No	18	8.1

Scores for attitude questions: A1. Unlikely–0, Neither likely nor unlikely—1, and Likely—2; A2. Take care at home—0, Avoid all physical contacts—1, and Take person to health facility—2; A3. Do not know/Not sure-0, Yes—1, No—2; A4. No—0, Do not know/Not sure—1, Yes—2; A5. Yes—0, No—1; and A6. Yes—1, No—0.

### Practices followed to prevent COVID-19 infection by hospital employees

**Figure 1:**
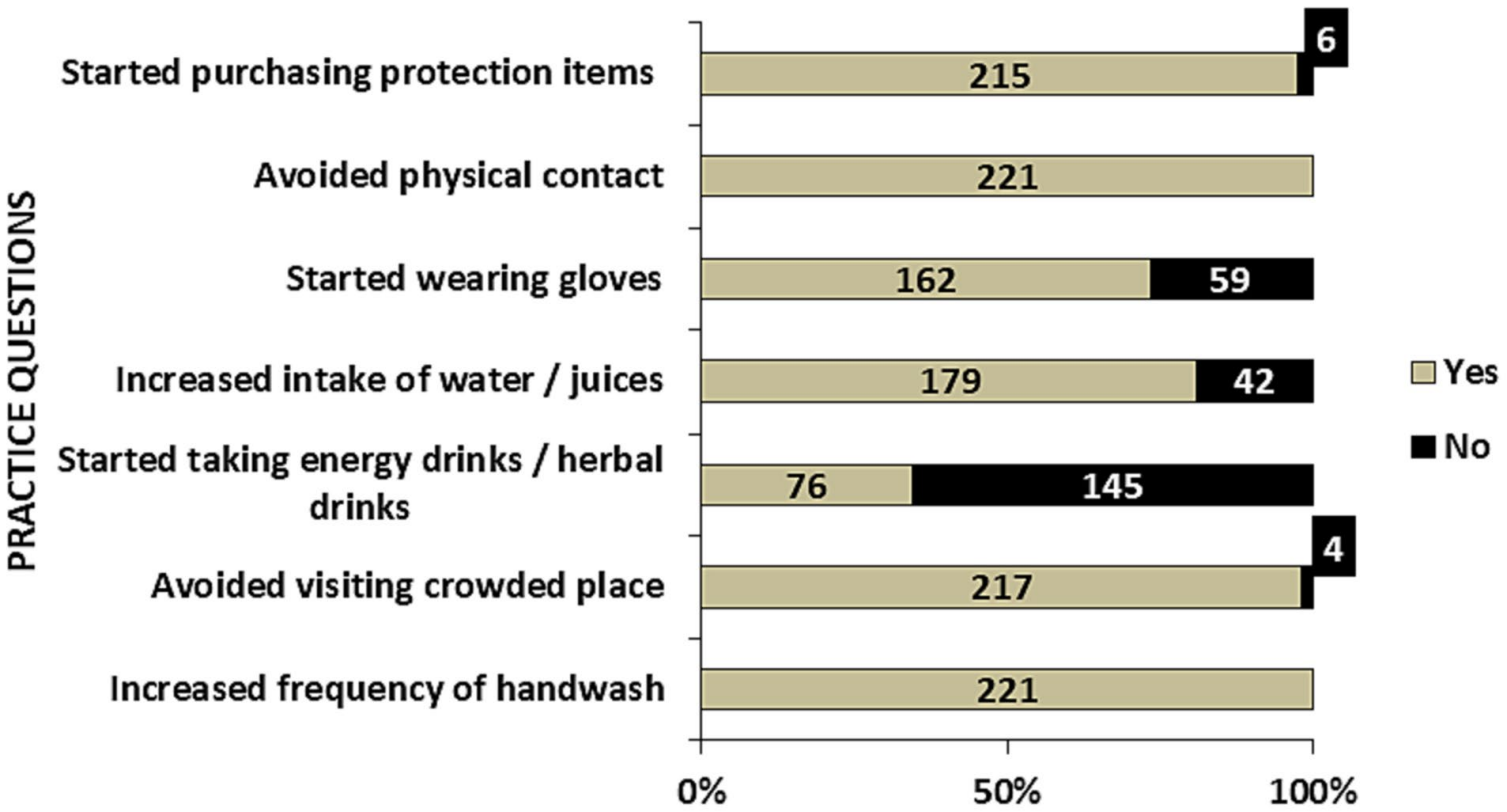
Distribution of practice questions responses in the study population.

The distribution of responses regarding prevention practices is shown in Figure 1. All the employees reported that their handwashing frequency had increased and that they were avoiding physical contact with others. This practice was reported to be followed by 145 (65.6%) employees for more than 3 months. Furthermore, (*n* = 179; 81.0%) employees reported having increased their intake of water or juices and 162 (73.3%) employees had started wearing gloves.

### Comparison of demographic characteristics and knowledge of COVID-19 infection, attitudes, and practices among hospital employees

The comparison of the level of knowledge, attitudes, and practices with the demographics and history of COVID-19 infection among the employees is shown in Table 4. Employees in the 31–50 age range showed higher knowledge scores for COVID-19 infection than younger and older employees. Similarly, non-Saudi employees had significantly higher knowledge scores compared to Saudi employees (7.08 ± 1.31 vs. 6.47 ± 1.70, respectively, *p* = 0.020). Moreover, physicians and employees with higher education levels had higher knowledge scores for COVID-19 infection than their counterparts. Non-Saudi employees had significantly higher attitude scores than their counterparts (7.49 ± 1.46 vs. 6.69 ± 1.73, respectively, *p* = 0.001). Non-Saudi employees and nurses had significantly higher levels of practice scores compared to their counterparts.

**Table 4:** Comparison of level of knowledge, attitude and practice with the demographics and history of COVID-19 infection of the participants.

Characteristics	Knowledge (Mean ± SD)		Attitude (Mean ± SD)		Practice (Mean ± SD)	
Age group						
21–30 (*n* = 68)	6.54 ± 1.52	*p* value = 0.018^*^	6.91 ± 1.46	*p* value = 0.098	5.72 ± 0.94	*p* value = 0.483
31–40 (*n* = 109)	7.06 ± 1.34		7.49 ± 1.64		5.88 ± 0.98	
41–50 (*n* = 34)	7.06 ± 1.43		7.44 ± 1.44		6.00 ± 0.85	
51–60 (*n* = 10)	6.60 ± 1.07		7.40 ± 1.35		5.70 ± 0.95	
Gender						
Male (*n* = 88)	7.02 ± 1.37	*p* value = 0.468	7.32 ± 1.55	*p* value = 0.907	5.69 ± 0.96	*p* value = 0.058
Female (*n* = 133)	6.88 ± 1.47		7.29 ± 1.56		5.94 ± 0.93	
Nationality						
Saudi (*n* = 51)	6.47 ± 1.70	*p* value = 0.020^*^	6.69 ± 1.73	*p* value = 0.001^*^	5.31 ± 1.09	*p* value = 0.0001^**^
Non-Saudi (*n* = 170)	7.08 ± 1.31		7.49 ± 1.46		6.00 ± 0.84	
Marital status						
Single (*n* = 78)	7.03 ± 1.48	*p* value = 0.443	7.18 ± 1.38	*p* value = 0.384	5.88 ± 0.97	*p* value = 0.619
Married (*n* = 143)	6.88 ± 1.40		7.37 ± 1.64		5.81 ± 0.94	
Children						
No (*n* = 95)	7.01 ± 1.47	*p* value = 0.506	7.18 ± 1.47	*p* value = 0.304	5.88 ± 0.93	*p* value = 0.563
Yes (*n* = 126)	6.88 ± 1.40		7.40 ± 1.62		5.80 ± 0.96	
Education level						
High school (*n* = 19)	6.26 ± 1.76	*p* value = 0.030^*^	6.79 ± 1.69	*p* value = 0.212	5.45 ± 0.98	*p* value = 0.095
Graduate (*n* = 178)	6.94 ± 1.37		7.31 ± 1.56		5.88 ± 0.94	
Postgraduate (*n* = 24)	7.42 ± 1.41		7.62 ± 1.41		6.00 ± 0.88	
Profession						
Non-medical (*n* = 45)	6.47 ± 1.44	*p* value = 0.010^*^	7.24 ± 1.69	*p* value = 0.323	5.37 ± 1.14	*p* value = 0.0001^**^
Para-medical (*n* = 22)	6.86 ± 1.21		6.95 ± 1.67		5.68 ± 0.99	
Nurses (*n* = 118)	6.82 ± 1.35		7.27 ± 1.35		6.16 ± 0.74	
Physicians (*n* = 36)	7.47 ± 1.23		7.69 ± 1.35		5.47 ± 0.91	
Have you tested positive for COVID-19 infection						
No (*n* = 210)	6.96 ± 1.42	*p* value = 0.252	7.28 ± 1.54	*p* value = 0.355	5.84 ± 0.94	*p* value = 0.933
Yes (*n* = 11)	6.45 ± 1.57		7.73 ± 1.79		5.82 ± 1.08	
Do any one who living with you in your home tested positive for COVID-19 infection						
No (*n* = 177)	6.90 ± 1.49	*p* value = 0.496	7.29 ± 1.56	*p* value = 0.858	5.84 ± 0.94	*p* value = 0.864
Yes (*n* = 44)	7.07 ± 1.17		7.34 ± 1.54		5.86 ± 1.00	
Have you self-quarantined or told to self-quarantine yourself						
No (*n* = 160)	6.79 ± 1.13	*p* value = 0.409	7.26 ± 1.60	*p* value = 0.530	5.86 ± 0.97	*p* value = 0.711
Yes (*n* = 61)	6.77 ± 0.99		7.41 ± 1.45		5.80 ± 0.89	

^*^p value < 0.05. ^**^p value < 0.0001.

## Discussion

The COVID-19 pandemic resulted in an increased burden of patients in hospitals. The work environment exposed all employees of the health institution to higher risks of contracting the infection. The present study illustrates the knowledge, attitudes, and practices of different types of employees working in a private health institution.

### COVID-19 knowledge level among different groups of hospital employees

The information regarding employees’ KAP about COVID-19 is important, as it will help in planning strategies to protect them during a crisis. The main findings of the present study are (i) employees demonstrated good knowledge of COVID-19 as they correctly answered the questions related to transmission (89.1%), and isolation period (87.8%), (ii) the age range of 31–50 years, non-Saudi nationals, those with higher education levels and physicians showed a significantly higher level of knowledge regarding COVID-19 infection. The present study’s findings suggest that healthcare institution might have focused on providing greater support and education programs for frontline workers than for nonmedical employees. An outbreak can be controlled efficiently if all employees know about the disease, which can be reflected in their attitudes and practices. According to McEachan et al. (23), knowledge is essential for building preventive beliefs, forming positive attitudes, and supporting positive behaviors. When a healthcare management team takes the lead as a role model and engages with employee groups in the face of difficult situations, the employees tend to prefer and embrace the practice followed by the team leader (24).

The present study’s results were in line with the findings of some studies (15, 16, 21), while some results conflict with those of other studies (13, 14). These differences might have arisen due to differences in the selection of study participants and the place of study. Bashir et al. (16) conducted a study among healthcare workers in the Riyadh region from March to April 2020 and reported that more than 90% of the participants had good knowledge about the symptoms of COVID-19, which is slightly higher than present study findings. Another study conducted from April 30 to May 14, 2020 reported that knowledge about the correct duration of handwashing was 63.7% among healthcare workers (18). Similarly, Almohammed et al. (14) reported that 67.8% of healthcare workers had adequate knowledge about COVID-19 and that a poor level of knowledge was associated with low education.

A study conducted among nurses in Saudi Arabia from April to May 2020 reported that 96.85% of nurses had excellent knowledge of COVID-19. The demographics associated with the higher level of knowledge were non-Saudi nationality, female gender, married, and a bachelor’s degree (13). A study conducted among paramedics in September 2020 reported that younger paramedics had sufficient knowledge. Further, they reported that the diploma holders had better knowledge than respondents with bachelor’s or master’s degrees (20). Alqarni et al. (21) conducted a study among employees of Al-Imam Abdulrahman Al Faisal Hospital in Riyadh from June to August 2020 and reported that male participants, employees in the 50–59 age group and paramedics had better knowledge about the treatment of choice available for COVID-19. Knowledge about transmission through direct contact with patients was higher among employees below 50 years of age. A cross-sectional study carried out from December 2020 to February 2021 reported that physicians had higher knowledge than nurses and pharmacists, but the difference was not statistically significant. Moreover, they reported that female participants, participants of Saudi nationality and those with a master’s degree had better knowledge scores than their counterparts (15).

### Attitude toward COVID-19 among different groups of hospital employees

The present study’s results showed that the employees had positive attitude toward vaccination for COVID-19 (91.9%) and 64.7% of employees agreed to inform the health facility if they suspected any family member had a COVID-19 infection. The results reflected a positive attitude and trust by the employees in the healthcare institutions regarding the care of their family members. Similar findings have been reported by some researchers. A study by Bashir et al. (16), conducted on healthcare workers, reported that 87.8% of the participants showed a positive attitude toward the COVID-19 pandemic. AlMohammed et al. (14) reported that 72.2% of healthcare workers had a positive attitude toward COVID-19 and that female participants had a better attitude compared to male healthcare workers, while nursing professionals showed less favorable attitudes. Similar results were reported by Al-Dossary (13), whose study population was nurses. Among them, 60.4% had a positive attitude toward COVID-19, and non-Saudi nurses had a higher positive attitude compared to their counterparts. Another study reported that those who were female, married and holders of bachelor’s degrees had a better attitude toward COVID-19. Similar to the nurses’ attitudes, a study conducted on paramedics also reported low scores for attitude (3.6 ± 0.6), with 42.3% of participants showing a positive attitude toward COVID-19 (20).

### Practices followed by different groups of hospital employees to prevent COVID-19

To control infection, along with good knowledge and a positive attitude, adequate preventive measures are necessary. In this study, it was found that the nurses’ scores for the practice of preventive measures were higher compared to the other employees. Nurses constitute the largest segment of hospital employees and have frequent direct physical contact with patients. Their better practices help in controlling the spread of infection in the hospital. If nurses neglect personal protection methods, they could increase the rate of infection among workers and patients (25).

All employees reported an increased frequency of handwashing to protect themselves from infection. This was similar to the findings reported by Bashir et al. (16), in which 91.49% of participants avoided a visit to crowded places and followed hygienic practices. Another study reported that 70% of healthcare workers strictly adhered to hand hygiene practices (18). AlMohammed et al. (14) reported that 80.2% of healthcare workers had good practice levels and nursing professionals applied best practices. However, a study conducted on paramedics reported a low practice score (1.9 ± 0.2), with 5.6% of the participants showing good practice (20). In terms of prevention and scientific control, a high value is placed on handwashing and wearing PPE. Workers must be aware when working in high-risk environments to limit the spread of infection.

## Conclusion

The study’s results revealed that the COVID-19 knowledge, attitudes, and practices of hospital employees are associated with their socio-demographic factors; level of education, nationality, and field of service. The study highlights that there is a gap in the level of knowledge about COVID-19, especially among nonmedical employees. The hospital administration should focus on providing intensive educational programs to all of its employees, including nonmedical employees, to promote affirmative preventive practice. Further, a targeted interventional programs need to be planned and implemented to improve COVID-19 awareness among non-medical employees.

### Limitations

The survey was conducted in a single private health organization, so the results may not apply to all health sector institutions. In the present study, a convenience sampling method was used, which might have resulted in selection bias. Moreover, it was a descriptive cross-sectional study in which a self-reporting questionnaire was used, which might have led to social desirability bias.

### Recommendations

Health institutions should emphasize regular workshops and training sessions, not only for medical and paramedical employees, but also for nonmedical employees. Education and training programs should be designed based on the level of education and knowledge needed. Additional efforts should be made to promote handwashing, as this is the most effective step in controlling the spread of infection. Measures such as hanging posters with the correct method and duration of handwashing should be displayed near wash basins throughout the hospital. Moreover, as employees have different levels of education and are of different nationalities, the message should be displayed not only in English and Arabic, but also in other languages used by the employees. This will motivate low-education employees providing nonmedical services to follow prevention measures effectively. This will also contribute to improving relations between employees and the administration.

## Data availability statement

The raw data supporting the conclusions of this article will be made available by the authors, without undue reservation.

## Ethics statement

The studies involving humans were approved by Institutional Research Board of Almoosa Specialist Hospital, reference number: ARC-20.12.06. The studies were conducted in accordance with the local legislation and institutional requirements. Written informed consent for participation was not required from the participants or the participant’s legal guardians/next of kin to avoid any physical contact with the participant during the pandemic, and the survey link was sent on mobile for self-administration of the questionnaire. In the first page of questionnaire, the aim of the study was written and a statement was included that participation is voluntary and personal information will not be disclosed. Verbal informed consent was obtained from all the participants. The study was performed in accordance with the Declaration of Helsinki.

## Author contributions

LA: Conceptualization, Data curation, Methodology, Writing – review & editing. NJ: Conceptualization, Formal Analysis, Supervision, Writing – review & editing. CC: Conceptualization, Writing – review & editing. HA: Data curation, Writing – original draft. BA: Data curation, Writing – original draft. MaA: Data curation, Writing – original draft. MC: Conceptualization, Methodology, Writing – review & editing. BB: Formal Analysis, Methodology, Writing – review & editing. MoA: Conceptualization, Writing – review & editing.
